# Exploration of the link between gut microbiota and purinergic signalling

**DOI:** 10.1007/s11302-022-09891-1

**Published:** 2022-09-19

**Authors:** MingJian Li, BoWen Liu, Rong Li, Ping Yang, Ping Leng, Yong Huang

**Affiliations:** 1 College of Medical Technology, Chengdu University of Traditional Chinese Medicine, Chengdu, 611137 China; 2 School of Pharmacy, Chengdu University of Traditional Chinese Medicine, Chengdu, 611137 China; 3 Innovative Institute of Chinese Medicine and Pharmacy, Chengdu University of Traditional Chinese Medicine, Chengdu, 611137 China

**Keywords:** Purinergic signalling, Gastrointestinal microbiome, Inosine, P2X receptors, A2A receptors

## Abstract

Growing evidence reveals that microorganisms in the gut are linked to metabolic health and disease risk in human beings to a considerable extent. The focus of research at this stage must tend to focus on cause-and-effect studies. In addition to being a component of DNA and RNA, purine metabolites can be involved in purine signalling in the body as chemical messengers. Abnormalities in purinergic signalling may lead to neuropathy, rheumatic immune diseases, inflammation, tumors, and a wide range of other diseases. It has proved that gut microbes are involved in purinergic signalling. The relationship between these gut-derived purinergic signalling molecules and host metabolism may be one of the important clues to our understanding of the mechanisms by which the microbiota affects host metabolism.

## Introduction

In recent years, multidisciplinary studies in epidemiology, pathology, histology, cells, and animals have uncovered a growing body of evidence that microbes in the gut are linked to metabolic health and disease risk in human beings to a considerable extent. Over the past 15 years, numerous descriptive studies have provided ample evidence for the theory that there is a bidirectional mechanism of influence between the gut microbiota and the metabolic homeostasis of the host, and that disordered gut flora contributes to the development of a variety of common metabolic diseases, including obesity, type 2 diabetes, nonalcoholic liver disease, metabolic heart disease, and malnutrition. To further understand the mechanisms of how the gut microbiota affects host metabolism, it is important to stand focus on cause-and-effect studies [1, 2].

Besides as a component of DNA and RNA, purine metabolites can also act as a chemical messenger to be involved in purine signalling in the organism, which can be cross-linked with other metabolic networks to coordinate many physiological processes such as cell proliferation, differentiation, migration, and apoptosis. Abnormalities in purinergic signalling can also lead to neuropathy, rheumatic immune diseases, inflammation, tumors, and many other diseases [3]. Adenosine 5′-triphosphate (ATP) is the most widespread purinergic signalling molecule, and this purinergic system exists in microorganisms in addition to mammals. Mitochondria are ATP-generating organelles, the endosymbiotic origin of which was a key event in the evolution of eukaryotic cells [4]; phylogenetic evidence suggests that alphaproteobacterial may be the ancestor of mitochondria, and mitochondria themselves are considered as a kind of alphaproteobacterial [5], although some studies have suggested that mitochondria evolved from a proteobacterial lineage that branched off before the divergence of all sampled [6]. However, evidence of microbial involvement in the purine system does exist, thus showing that microorganisms are inextricably linked to the purine system.

It has been shown that intestinal microorganisms can release extracellular ATP (eATP) into the gut or metabolize it to produce adenosine-like derivatives. The eATP from intestinal flora is able to bind to P2X7 receptors on small intestinal Tfh cells to maintain host immune homeostasis [7], and the adenosine metabolites from intestinal microbes can participate in the immune regulation via A_2A_ receptors in the gut [8, 9]. The relationship between these gut-derived purinergic signalling molecules and host metabolism may be one of the important clues to our understanding of the mechanisms in which the microbiota affects host metabolism.

This paper aims to summarize the evidence that gut microbes are involved in the purinergic system and to discuss the possible causal relationships. The combining purinergic systems have provided a new way to study the gut microbiota. The studies in the metabolism of the gut microbes and purinergic systems have demonstrated their respective roles in a variety of metabolic diseases, immune regulation, tumors, and microbial-derived purinergic signalling. This provides the potential to serve as a bridge linking the microbiota, purine metabolism, and various physiological phenomena or disease occurrence.

## Purinergic system

Cellular metabolism involves a highly regulated series of sequential biochemical reactions designed to produce the necessary substrates for essential cellular processes. Purines are one of the most abundant metabolites in mammalian cells. In addition to the production of DNA and RNA molecules, purine nucleotides such as adenosine 5′-triphosphate (ATP) and guanosine 5′-triphosphate (GTP) are essential for the provision of cellular energy and intracellular signalling, respectively. Purines can also be incorporated into more complex biomolecules and act as cofactors, such as nicotinamide adenine dinucleotide (NAD) and coenzyme A. Purine metabolites are involved in energy transfer and nucleotide metabolism in the body, providing essential energy and cofactors for cells, and are important basic metabolic compounds in the body.

The metabolic environment in which the intracellular purine metabolites are interconverted together constitutes the cellular purine pool. Intracellular adenosine can be converted to AMP by phosphorylation and thus reused by the body, and AMP and other purine nucleotides (i.e., inosine monophosphate (IMP), xanthosine monophosphate (XMP), and guanosine monophosphate (GMP)) can be interconverted to form intracellularly, resulting in a cellular purine pool. In addition, bases present in the extracellular matrix can be translocated into the cell to produce the corresponding nucleotides.

Uric acid (UA) salts are the end product of intracellular purine metabolism. The catabolism of purine nucleotides takes place mainly in the liver, small intestine, and kidney. The accumulation of UA can result in hyperuricemia (HUA) and the deposition of monosodium urate (MSU) in the joints can cause gout, which is associated with dysregulation of purine catabolism [10]. Diets rich in purines (including seafood, meat, animal offal, and alcohol) can lead to excessive UA production [11]. The metabolism of alcohol and fructose consumes a large amount of ATP, which leads to the accumulation of AMP and thus accelerates the breakdown of nucleotides and increases the production of UA [12]. UA is produced through the oxidation of xanthine by xanthine oxidase; the use of xanthine oxidase inhibitors can alleviate this metabolic abnormality [13]. The kidneys and intestines are responsible for the UA excretion and about 25% of UA is excreted into the intestines [14]. Recent evidence suggests that the pathogenesis and progression of HUA are inextricably linked to gut microbes [15]. The intestinal bacteria such as *Escherichia coli*, *Clostridium*, and *Pseudomonas* can be involved in the metabolism of UA in the intestine [16, 17]. Enrichment of *Bacteroides caccae*, *Bacteroides xylanisolvens*, and other flora could be detected in the feces of gout patients, while there was a decrease in the abundance of genera such as *Faecalibacterium prausnitzii* and *Bifidobacterium pseudoatenulatum* [18]. Meanwhile, the transplantation of feces from HUA mice into normal mice resulted in an increase in UA levels in normal mice [19]. All of this evidence suggests a link between purine metabolism and gut microbes.

## Possible targets of gut microbiota involved in purinergic signalling

In addition to direct involvement in purine metabolism, gut microbes can also participate in the regulation of metabolism in vivo through purinergic signalling. This regulatory effect is mainly mediated through purinergic receptors in the intestine and the release of extracellular ATP (eATP) and nucleoside metabolites from gut microbes, which is also influenced by extracellular nucleotidases.

Apart from participating in the synthesis of DNA and RNA, purine metabolites can also bind to purine receptors directly or as ligands for purinergic reactions, allowing purine metabolites to participate in cellular communication as a signalling molecule. Extracellular nucleotides (e.g., ATP, UTP, ADP, NAD) and their derived nucleosides (e.g., adenosine, inosine) are initiators and major components of purinergic reactions [20]. Purinergic signalling can regulate many aspects of cell behavior, such as proliferation, differentiation, migration, apoptosis, and other important cellular physiological processes, through synergistic interactions with other transmitter networks. Purinergic receptors on intestinal epithelial cells as well as immune cells inevitably have a complex connection with the gut microbiota. The microbial ecosystem is an important player in the treatment and intervention of inflammatory bowel disease and some autoimmune diseases, which makes the link between gut microbes and purinergic signalling potentially harboring important physiological implications.

Purinergic receptors are divided into two subfamilies: P1 receptors and P2 receptors. The P1 receptor is a G protein–coupled receptor, and adenosine can act as an endogenous ligand to bind to the P1 receptor and participate in physiological responses such as cardiac rhythm regulation [21]. The P1 receptor family has four main members: A_1_, A_2A_, A_2B_, and A_3_. A_1_ receptors are mainly expressed in the nervous system, while the other three subtypes are widely expressed in the nervous system, spleen, colon, testis, and other tissues [22].

The pathophysiological function of P1 receptors is extremely complex. A_1_ and A_3_ receptors can bind to Gi proteins and reduce intracellular cyclic AMP (cAMP) levels by inhibiting adenylate cyclase (AC) activity. In contrast, A_2A_ and A_2B_ bind to Gs proteins and activate AC to upregulate intracellular cAMP levels [23]. CD8^+^ T cells predominantly express A_2A_ and A_2B_ receptors. Activation of A_2A_ receptors increases intracellular cAMP levels, inhibits Th1/Th2 differentiation, and increases intracellular cAMP levels, exerting an immunosuppressive effect during immune regulation [24]. In addition, A_1_ and A_2A_ receptors (A_2A_R) can form A_1_-A_2A_ complexes that bind to both Gi and Gs proteins and can exert opposite regulatory effects depending on the ligand concentration [25, 26]. The use of A_2A_R activators reduces symptoms in mice with colitis, which clarifies the link between A_2A_R and colitis [27]. Gut microbiota–mediated modulation of A_2A_R signalling alleviates DSS-induced intestinal inflammation model and simultaneously detects an increase in plasma inosine concentration in mice [28]. This suggests that the activation of A_2A_R may be a pathway to reduce the symptoms of colitis and that intestinal microbes are involved in this metabolic process.

The P2 nucleotide receptor family is divided into two subfamilies: P2X and P2Y [29]. P2 receptors can be activated by ATP and its analogs to regulate cellular metabolism and influence inflammatory and immune responses. Extracellular ATP can directly regulate T cell responses and induce intestinal inflammation by binding to P2X receptors [30]. Apart from the release of extracellular ATP from the damaged cells, intact cells also release ATP for the regulation of various immune cell functions such as the maturation of dendritic cells (DCs) and the activation of B and T cells under normal conditions [31]. P2 receptor has received a great deal of attention as a therapeutic target for colonic inflammation. It was found that P2RX1 knockout (P2rx1^−^/^−^) mice were less affected by DSS compared to WT mice. Meanwhile, the indole alkaloid biogenesis pathway was upregulated, and indole-producing microbes such as *Clostridiaceae*, *Bacteroidaceae*, *Rikenellaceae*, and *Lachnospiraceae* families were enriched in the intestine [32]. Indole alkaloids have been shown to induce regeneration of the mucosal barrier and to play a protective role in colitis [33]. Such results suggest that P2RX1 knockout–induced changes in gut microbiota could provide stronger gut protection. The cause of this phenomenon needs to be further confirmed.

Extracellular ATP can be catabolized by extracellular nucleotidases (e.g., CD39, CD73) to ADP and AMP, eventually generating adenosine (ADO) to transmit purinergic signals between cells. CD39 is the prototype of the ectonucleoside triphosphate diphosphate hydrolase (ENTPDase) family. ENTPDase1/CD39 can be expressed in immune cells and regulate the immune response by downregulating ATP levels. ENTPDase7 is the major ectonucleoside triphosphate diphosphate hydrolase in the intestine. The *Entpd7* gene, which encodes ENTPDase7, is highly expressed in all types of intestinal epithelial cells (EC) in the small intestine. The expression of *Entpd7* was not altered in the small intestine of mice treated with oral antibiotics, indicating that the microbiota did not directly regulate the expression of *Enptd7* [34].

ENTPDase8 is highly expressed in large intestinal epithelial cells and can regulate eATP concentration in the large intestine [35]. In fecal microbiota of P2rx4^−^/^−^ mice, the relative abundance of *Bacteroidetes* was greater than that in WT mice, while the relative abundance of *Proteobacteria* was lower [35]. These results suggest that P2X4R contributes to the maintenance of the gut microbial community. It has also been found that ENTPDase8 can similarly reduce intestinal inflammation by limiting P2Y6 receptor activation [36]. But additional studies on the P2Y6 receptor have found that P2Y6^−^/^−^ mice are more susceptible to DSS-induced inflammation than WT mice, and P2Y6 deficiency can affect the secretion of a protective mucus layer in the gut or lead to abnormal activation of Th17/Th1 lymphocytes [37, 38]. This change may be related to the alteration of intestinal microbiota after P2 receptor deletion, and high-affinity IgA may maintain the balance of intestinal ecology by limiting the growth of certain bacteria. More studies are needed to clarify this overall regulatory effect.

Extracellular ATP is usually considered to be a pro-inflammatory signal, but extracellular adenosine produced by ATP/ADP/AMP degradation exhibits immunosuppressive effects. Thus, production by adenosine can often exert a regulatory effect on the eATP-induced inflammatory response [39]. Some evidence suggests that the gut microbiota can regulate the purinergic signalling by releasing eATP or by participating in nucleotide metabolism in which this connection may play an important role in purinergic signalling–mediated immunomodulatory effects [32, 40–44]. In the following, we will further discuss the possible mechanisms of this moderating effect.

## Possible mechanisms of extracellular ATP from commensal bacteria involved in purinergic signalling in the intestine

The intestinal microbiota is involved in the formation of the intestinal mucosal barrier to maintain the homeostasis of the intestinal environment and can participate in purinergic signalling in the gut by releasing eATP to bind to P2X7 receptors in the intestine. This mechanism can maintain the homeostasis of microflora in the intestine through immunomodulatory effects. Several studies focusing on the P2X7 receptor (P2RX7) on T follicular helper (Tfh) cells in Peyer’s patches (PPs) have shown that the microbiota in the gut can release ATP via mechanosensitive channels and the eATP may be involved in purinergic signalling through the gut, affecting a range of inflammatory responses and immune regulation [45, 46].

Microbes in the gut can stimulate the development of gut-associated lymphoid tissue [47], Tfh cells in PPs, a subset of CD4^+^ T cells, promote germinal center (GC) responses and IgA release, thereby limiting the colonization of invasive bacteria and the entry of microbial-associated inflammatory factors from the intestinal lumen, constituting an effective mucosal defense mechanism in the intestine [48, 49]. High-affinity IgA protects the intestine by limiting bacterial growth [50], and the regulation of the high-affinity IgA response by T follicle–regulated (Tfr) cells promotes intestinal flora diversification and influences the composition of the intestinal flora [51]. It has been found that ATP released by bacteria can penetrate the intestinal epithelium and affect intestinal homeostasis by inducing apoptosis of the cells that regulate high-affinity secretory IgA in the intestine. High concentrations of ATP can be found in ileal and hepatic portal blood. Vancomycin/ampicillin/metronidazole (VAM) treatment leads to a significant increase in ATP concentrations in ileal and hepatic portal blood and induces massive apoptosis of the cells, a phenomenon that suggests that antibiotic treatment may harm the induction of this intestinal immunity [52]. In contrast, depletion of eATP in the intestine with adenosine triphosphate bisphosphatase (apyrase) enhances the sIgA response. In addition, the addition of apyrase to oral vaccine formulations against bacteria to counteract eATP in the intestine has been used to boost IgA titers in serum [52]. All these phenomena demonstrate the association of eATP with gut microbiota homeostasis.

The P2RX7 purinergic receptor is an ATP-gated cation channel to which high concentrations of extracellular ATP bind to activate the NLRP3 inflammasome pathway, ultimately leading to the maturation and release of the pro-inflammatory cytokines IL-1β and IL-18 [53, 54]. P2RX7 plays a key role in the innate immune response and regulation of the T cell population [55, 56]. In the intestines, the activation of P2RX7 downregulates the number of Tfh cells in PPs to promote host-microbiota symbiosis under homeostatic conditions [40], and drives Th1 cell differentiation and controls follicular helper T cell populations to prevent Plasmodium malaria [57]. Mice lacking P2X7 (P2rx7^−^/^−^) exhibited a significant increase in Tfh cells due to impaired P2XY receptor function, resulting in resistance to eATP-induced apoptosis. The alteration of Tfh cells in these mice resulted in an enhanced secretory IgA response [40]. This suggests that beyond its singular pro-inflammatory role, P2RX7 regulates the intestinal environment in a more complex manner.

Hierarchical clustering of mouse cecum microbiota revealed an increase of *Lachnospiraceae* and *Helicobacteraceae* in the intestine of P2rx7^−^/^−^ mice [7]. In contrast, enrichment of *Paraprevotellaceae* and *Caulobacteraceae* occurred in wild-type mice [7]. The increase in *Lachnospiraceae* may be associated with obesity [58]. Many species of this genus have been shown to produce butyrate [59]. The occurrence of these microbiome changes may be due to the deficiency of P2RX7 [7]. This shows that blocked purinergic signalling can directly affect the composition of intestinal microbes, which demonstrates the complex link between purinergic signalling and intestinal microbes in the intestine. Lack of P2RX7 leads to an increase in PP Tfh cells, but not in the cellular abundance of Tfr. Dysregulation of Tfh cell activation may lead to a loss of controlled diversity of stimulatory bacteria that play an important role in the regulation of gut microbial homeostasis [51].

In addition, the intestinal microbiota can assist in the constitutive development of Th17 cells in the intestinal lamina propria (LP). The segmented filamentous bacteria (SFB) are among the most potent Th17 cell inducers [60]. SFB are a mucosa-associated symbiont in the intestine composed of a group of spore-forming Gram-positive bacteria. Close adhesion to small intestinal epithelial cells (EC) is a distinctive feature of SFB [61], and the colonization of SFB can trigger unique signalling pathways in the intestine to produce a favorable environment for Th17. There is evidence that microbiota-mediated Th17 cell development is independent of the major innate immune receptors [62]. SFB as a component of the gut microbiota can promote autoimmune arthritis by inducing PP Tfh cells, and SFB-containing mouse feces can exacerbate the inflammatory response in SPF K/BxN mice, but there is evidence that the immune response induced by SFB is not directly linked to the role of P2RX7 [63]. Notably, microbial-host interactions were also observed in P2RX7 deficiency concerning host-influenced microbiota. For example, it has been previously shown that P2RX7 deficiency triggers an enhanced PP Tfh cell response, leading to increased IgA production by B cells to inhibit SFB colonization [40].

## Adenosine derivative in the intestine involved in immunomodulatory effects

Gut microbes can also regulate the activity of A_2A_R by affecting the level of inosine, thus participating in a complex immunomodulatory process. Inosine is formed from adenosine in a reaction catalyzed by adenosine deaminase in the intracellular and extracellular spaces. IMP is dephosphorylated intracellularly to inosine by 5′-nucleotidase and shunted to the extracellular space via the membrane nucleoside transporter. Inosine is degraded intracellularly by purine nucleoside phosphorylase to hypoxanthine and ribulose 1-phosphate. Hypoxanthine is subsequently converted to xanthine catalyzed by xanthine oxidase and eventually converted to UA [64].

In the past, inosine was generally thought to be an inactive breakdown product of adenosine, but it has since been shown that inosine can also bind to adenosine receptors and initiate intracellular signalling. Among the four AR subtypes, A_2A_R is the most effective in downregulating inflammation by regulating intracellular cAMP levels, but inosine is thought to be a functional agonist for A_1_R and A_3_R. Recent evidence has also supported the active effect of inosine against A_2A_R [65]. The theoretical basis for the belief that inosine is not an effective agonist of A_2A_R is mostly derived from in vitro experiments [66, 67]. In contrast, in experiments in vivo, inosine tends to exert A_2A_R-dependent anti-inflammatory and immunomodulatory effects [67]. With the advancement of gut microbial studies and probiotic-related studies, the link between the biological activity of inosine and microorganisms provides more perspectives on the biological significance of inosine.

*Lactobacillus reuteri* strain DSM 17,938 (LR) is a probiotic originally isolated from breast milk [68]. LR has been shown to have anti-inflammatory and immunomodulatory effects by inhibiting the Toll-like receptor 4-mediated NF-κB pathway [69]. In recent years, it has been found that its mechanism of action is related to purinergic signalling and inosine is a key effector molecule in *Lactobacillus* roxellanae therapy. Studies have found that LR can alleviate autoimmune diseases caused by defective regulatory T (Treg) cells by restoring inosine levels in the intestine [41, 70]. Forkhead box protein 3 (Foxp3) is a major transcription factor associated with T reg cell development and function. Mutations or deletions of the Foxp3 gene cause human IPEX syndrome (immune dysregulation, polyendocrinopathy, and enteropathy, with X-linked inheritance), an autoimmune disease associated with Treg deficiency. Scurfy (SF) mice with mutations in the Foxp3 gene can exhibit a similar clinical phenotype [71].

Treg deficiency led to an increase in the number of IFN-γ-producing CD4^+^ T cells (Th1) and IL-4-producing CD4^+^ T cells (Th2) in mouse spleen MLNs and significantly reduced the alpha diversity of the intestinal flora in SF mice, while the relative abundance of *Lactobacillus* was significantly reduced and the relative abundance of *Bacteroides* was significantly increased in the feces of SF mice [8].

In contrast, oral administration of LR reversely reduced the plasma levels of IFN-γ and IL-4 in SF mice and prolonged the survival time of SF mice. Meanwhile, the relative abundance of *Lactobacillus*, *Oscillospira*, and *Firmicutes* was increased while that of *Tenericutes* and *Bacteroides* was decreased in the feces of SF mice treated with LR [8], but the effects of these alterations in the metabolic process still need to be confirmed by more studies. The in vitro fermentation showed that LR could not metabolize inosine directly, but LR treatment can restore normal plasma levels of inosine, which may be related to the upregulation of expression of equilibrative nucleoside transporter 1 (ENT1) and concentrative nucleoside transporter 2 (CNT2) in the intestine, assisting the entry of inosine in the intestinal lumen into the intestinal epithelium [72, 73]; the altered gut microbiota composition may also have influenced this process, but the exact mechanism of this action needs to be identified by additional studies. Undeniably, A_2A_ receptors (A_2A_R) play a key role in the process of remodeling the gut microbiota and suppressing autoimmune responses that occur due to Treg cell defects; the use of A_2A_R antagonists directly counteracts the immune-protective effects induced by LR [74].

Inosine has immunomodulatory effects on immune cells such as macrophages [64] and also functions as an immunosuppressive agent by binding to adenosine A_2A_R, thereby stimulating the release of intracellular cyclic adenosine monophosphate (cAMP) and inhibiting Th1/Th2 differentiation [8, 64]. Thus, the microbiota-inosine-A_2A_R axis can be an alternative approach to perform immunomodulatory functions similar to those of Treg cells when Treg cells are functionally deficient and unable to play a normal immunomodulatory role, which provides a potential pathway for the gut microbiota to adjust the immune function of the organism.

Besides, several intestinal bacteria were found to be associated with the efficacy of immune checkpoint blockade (ICB) [9, 44]. By using colorectal cancer (CRC) animal models to identify specific ICB-promoting bacteria, the researchers found that inosine produced by specific gut bacteria *Bifidobacterium pseudolongum* (*B. pseudolongum*) or *Akkermansia muciniphila* (*A. muciniphila*) and *Lactobacillus johnsoni* (*L. johnsonii*) can combine with adenosine A_2A_R to enhance the immunotherapeutic response and reduce the incidence and the size of inflammation-induced colon cancers in mice [9].

Oral supplementation of *B. pseudolongum* to germ-free mice with MC38 colon cancer enhanced the effect of immunotherapy blocked by CTLA-4 or PD-1, the process in which enhanced activation of Th1/Tc1 cells and their corresponding effector functions were observed. Further investigation of the mechanism of this phenomenon revealed that inosine produced by bacterial metabolism and A_2A_R on T cells are critical factors in this process. Compared with germ-free mice, mice treated with CTLA-4 blocker plus *B. pseudolongum* had increased serum inosine and its degradation factors xanthine and hypoxanthine, and inosine concentrations were highest in the duodenum and gradually decreased along the small and large intestine. This antitumor activity can be mimicked by the use of dibutyryl cAMP (Bucladesine, db-cAMP), which was depleted by either blocking A_2A_R or purging inosine [9].

*A. muciniphila* can modulate PD-1 blockade and produce inosine in vitro in an interleukin 12–dependent way by increasing the recruitment of CCR9^+^ CXCR3^+^ CD4^+^ T lymphocytes to the mice tumor bed [75]. The potentiation of ICB efficacy by *A. muciniphila* is also based on the A_2A_R expression of T cells [9, 75]. *L. johnsonii* promotes the antitumor efficacy of anti-CTLA-4, and its metabolite hypoxanthine also acts as a ligand binding to A_2A_R on the surface of T cells, though the ICB-promoting effect induced by *L. johnsonii* is relatively weak. This effect of enhanced immune activity is in contradiction with the function of A_2A_R in ordinary physiological conditions, which, as mentioned above, exerts immunosuppressive effects on inhibiting Th1 differentiation through the release of cAMP [9].

Novel immune checkpoint inhibitors based on the immunosuppressive effects of adenosine and A_2A_R signalling are also available, such as monoclonal antibodies (MAb) targeting CD73, CD39, and CD38, as well as pharmacological antagonists of A_2A_R, many of which are currently in clinical trials [76]. However, some studies have shown that inosine analogs can play a pro-inflammatory role, and A_2A_R can maintain Th1/anti-tumor immunity in mice [77–79].

This phenomenon may be related to the influence of the specific environment on which inosine is dependent when it is engaged in immune regulation.

In vitro experiments showed that inosine promotes Th1 differentiation of T cells when exogenous IFN-γ is present and, conversely, inhibits Th1 differentiation when IFN-γ is absent. cAMP is a downstream signalling molecule of A_2A_R. In the condition of blocking A_2A_R, db-cAMP can restore Th1 differentiation circumventing the requirements of inosine [9]. Moreover, even in the absence of exogenous IFN-γ, inosine could equally enhance Th1 differentiation when naïve T cells are triggered by anti-CD3/anti-CD28. Thus, the regulatory impact of the inosine-A_2A_R pathway on Th1 is affected by the specific microenvironment in which it is exposed, acting either as a promoter or as a suppressor [9].

The combination of inosine compounds coupled with CTLA-4 blockade alone was unable to exert antitumor activity in mice, while the addition of the immune adjuvant synthetic oligodeoxynucleotides that contain unmethylated CG dinucleotides (CpG ODN) resulted in the recurrence of the antitumor activity exhibited by *B. pseudolongum* plus CTLA-4 blockade exhibited reproducible antitumor activity, demonstrating the reliability of A_2A_R on the microenvironment [9]. Gut microbes are linked to the body’s immune system through complex and diverse pathways, and studies about immunomodulation through optimization of the gut microbiota remain to be deepened, and that these discoveries based on purinergic signalling may provide brand new insights for future studies.

## Dietary and herbal interventions associated with gut microbial–mediated purinergic signalling

The effects of diet can also affect purine metabolism and purinergic signalling through the involvement of gut microbes. It has been found in past studies that inappropriate dietary habits can lead to disturbances in purine metabolism, often culminating in the accumulation of purine metabolites leading to diseases such as HUA and gout [10], accompanying changes in gut microbiology [80]. High fructose intake exacerbates purine degradation and intestinal ecological dysbiosis [81–83], and a high-purine diet can also lead to disturbances in the intestinal ecology [19, 84]. Dietary and herbal interventions can play a useful part in the early prevention or treatment of disease, and if their mechanisms of action are clarified, they can also advance the development of targeted drugs in the future.

There has been much evidence that natural products can alleviate HUA by modulating the gut microbiota, alleviating chronic inflammation, and promoting UA excretion [85], as well as inducing microbial-associated purinergic signalling to improve the symptoms of related diseases. Rhein is the main component of several traditional Chinese medicines, including Rhubarb, Aloe, and Sennae Folium. Rhein treatment alleviated DSS-induced colitis, altered gut microbiota composition, and increased Lactobacillus level. It also alters the purine metabolism level to restore the concentration of UA. As Rhein is unable to metabolize UA directly, this regulatory effect may be related to the enrichment of *Lactobacillus* [86]. Sunflower head enzymatic hydrolysate (SHEH) can ultimately alleviate HUA by restoring disturbances in the composition of the gut microbiota and reducing circulatory LPS levels [87], a *Dendrobium candidum* leaf extract was also reported to have a similar function [88].

There is already some evidence that certain active ingredients from dietary fiber or Chinese herbs can participate in purinergic signalling regulation via the gut microbiota. Cordycepin (CCS) is a major bioactive component separated from Cordyceps militaris. Its chemical identity is 3-deoxyadenosine. CCS was found by improving gut microbiology in high-fat diet (HFD) mice and regulating body weight by upregulating adenosine A1 receptor expression and inhibiting prolactin secretion [89, 90]. But its specific targets of action need to be further defined. Barley leaf (BL) is the young grass of the crop barley (Hordeum vulgare L.). Its antioxidant properties have been reported in previous studies [91]. As a traditional Chinese medicine, it has been used to protect the function of the intestinal tract. The study found that, in addition to improving dysbiosis of the intestinal microbiota induced by dextran sodium sulfate (DSS), BL significantly affected the levels of purine metabolites in serum and colonic contents, with the most significant increases in inosine and guanosine in vitro. The fermentation experiments revealed that BL could be used by upregulating the relative abundance of *Firmicutes* and *Lactobacillus* and that enriched *Lactobacillus* was positively correlated with the concentrations of inosine and guanosine. Subsequently, further studies revealed that inosine could exert a protective effect against colitis through A_2A_R/PPARγ signalling [28]. This provides a basis for the involvement of dietary interventions in the regulation of purinergic signalling through gut microbes.

Inosine and guanosine are valuable food additives which can be manufactured by microbial fermentation techniques in industrial production. By using genetic engineering, several bacterial strains such as *Bacillus subtilis* [92], *Corynebacterium ammoniagenes* [93], and *Escherichia coli* [94] have been used to increase the production of these purine nucleotides.

In recent years, engineered yeasts developed through biosynthetic techniques have also found novel applications in the regulation of purinergic signalling mediated by the gut microbiota [95, 96]. Based on an inflammatory therapeutic strategy that regulates eATP-adenosine homeostasis, Benjamin M. et al. developed a novel engineered yeast; this engineered yeast can secrete apyrase in response to the metabolite eATP produced in the inflamed gut, thereby depleting pro-inflammatory eATP and promoting the production of immunosuppressive adenosine [43]. The use of engineered yeast probiotics to modulate the pro-inflammatory-anti-inflammatory balance in the gut may provide a more flexible treatment for intestinal inflammation and inflammatory diseases targeting tissues other than the intestinal system. All of this evidence suggests that probiotics can indeed act on the gut microbiota and that natural medicines and probiotics can indeed be involved in metabolic or disease processes through inflammatory and immune responses by participating in the metabolism of gut microbes.

## Conclusion

**Fig. 1:**
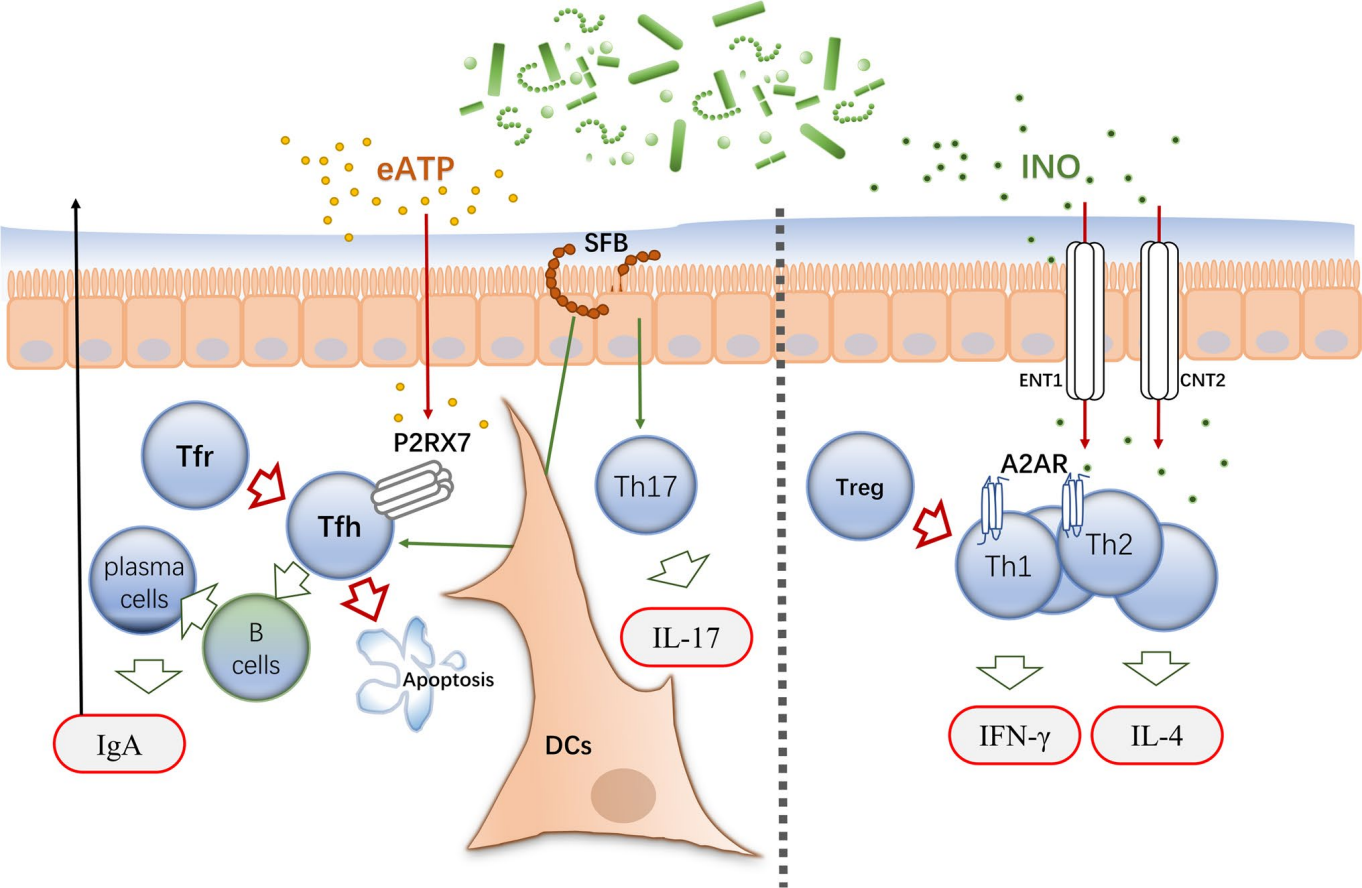
The release of eATP from the gut microbiota can induce apoptosis of Tfh cells and regulate IgA release by binding to P2X7 receptors on Tfh cells in Peyer’s patches (PPs). Also, the intestinal commensal SFB can induce the differentiation of Th17 and Tfh and stimulate the release of IgA with other cytokines. When the intestinal environment is stable, IgA produced in PPs can play a protective role in the intestinal environment. In contrast, once the homeostasis of the intestinal environment gets disrupted, SFB induces a strong autoimmune response. Inosine (INO), which is metabolized by some microorganisms in the gut, can act as a purinergic signalling factor binding to A2AR on the surface of CD4^+^ T cells, inhibiting the differentiation of helper T cells to play a Treg-like role. However, in a tumor environment or the presence of other inflammatory factors infiltration, the inosine-A2AR axis can play the opposite role and promote T cell differentiation

Over the last fifteen years, great progress has been made in the human microbiota field, yet difficulty lies in figuring out the specific functions performed by each microbial species in the prognosis or treatment of human diseases and in identifying the effective biomarkers that play key roles. By summarizing the existing studies, we suggest that ATP and adenosine derived from gut microbes can transmit purinergic signals in vivo by entering the gut as well as the bloodstream and binding to the corresponding purinergic receptors (Fig. 1).

Gut microbes can either actively release ATP into the gut or passively release ATP into the gut lumen after being disrupted. These extracellular ATPs can induce apoptosis of Tfh cells by binding to P2X7 receptors in Peyer’s patches (PPs), thus exerting immunosuppressive effects. And the activation of Tfh cells stimulates plasma cell differentiation and production of high-affinity SIgA to kill invasive pathogens in the intestine. This immune regulation modulated by microbial-derived eATP can maintain the stability of the gut microbiome that shows a form of co-existence between host and microorganism. Evidence suggests that immune responses mediated by SFB may also be related to this mechanism. The SFB, as a gut symbiont, contributes to immune homeostasis by inducing Th17 cells in normal physiological metabolism and inducing a strong autoimmune response when the gut microbiota is in an ecological imbalance. SFB can invoke Th17 cells to differentiate and produce multiple immune factors, leading to many autoimmune diseases, including rheumatoid arthritis (RA). Conducting more studies linking alterations in gut microbes with eATP and related purinergic receptors could provide more evidence for SFB-related metabolic mechanisms. Infection or immunity induces Tfh cells to produce complex immune responses, the disruption of Tfh cells can also alter the intestinal commensal community, and dysbiosis of the microbiota in the gut can stimulate Tfh cells to participate in immune regulation, emphasizing the importance played by Tfh cells in intestinal immune homeostasis. Immunomodulatory signals from the gut play a different role, and there may be additional applications for the immunosuppression exerted by P2X7 receptors in intestinal immunity beyond the multiple immune stimuli. Aiming to clarify the specific mechanism of this immunoregulation, more in vitro experiments on P2X7 receptors are needed.

In addition to eATP signalling, the adenosine derivatives inosine and hypoxanthine produced by microbial metabolism can also combine to A_2A_R on T cells and induce or inhibit Th cell differentiation in different contexts, thereby participating in the bidirectional regulation of the immune response. When Treg cell functionality is defective, the microbiota-inosine-A 2A receptor axis can exert immunomodulatory functions similar to those of Treg cells. For example, including *B. pseudolongum*, several bacteria promote the mouse immune system in tumor environment by inducing Th1 differentiation by the combination of their inosine-metabolites and A_2A_R, thereby enhancing the antitumor activity of ICB therapy. This phenomenon contradicts the immune-suppressive activity previously demonstrated by A_2A_R. In vitro experiments confirm that A_2A_R requires the coactivation of IFN-γ or CpG to exert this immune-promoting effect. The context-dependent feature shows the complex mechanisms behind the purinergic signalling–related immune regulation and highlights the necessity of a multi-omics study on it. Adenosine is the main ligand of A_2A_R, but metabolomic studies revealed extremely low levels of adenosine in the intestinal tract, while high concentrations of inosine are involved in purinergic signalling, which may be implicated in the metabolic reaction occurring in the intestine. To determine whether adenosine is a gut-derived purinergic signalling molecule required more microbiome as well as metabolomic studies. LR can improve plasma inosine concentrations but does not produce inosine in the in vitro experiments, and its direct link to metabolites needs to be further explored. This similar regulatory mechanism may also not be specific to LR, and there is much room for exploration of the link between microbial and purinergic signalling.

Different gut microbes can play a role in facilitating or inhibiting the immune response in the gut through diverse pathways. The maintenance or restoration of this homeostasis could be of great value in the development of gut flora–based therapeutic regimens. Existing studies have shown a close link between the purinergic system and gut microbial metabolism, but further microbiomic and metabolomic studies need to be combined with multifaceted analyses to clarify this underlying link. In addition to affecting the metabolism of uric acid, these effects also play an important role in the regulation of purinergic signalling. The discovery of more specific links between gut microbes and purinergic signalling could provide a more theoretical basis for the development of microbiota-based therapeutic approaches, as well as a detailed explanation of the physiological functions of purinergic signalling.

## Data Availability

Data sharing is not applicable to this article as no datasets were generated or analyzed during the current study.

## Abbreviations

ATP: Adenosine 5′-triphosphate
eATP: Extracellular Adenosine triphosphate
GTP: 5′-Triphosphate
NAD: Nicotinamide adenine dinucleotide
IMP: Inosine monophosphate
XMP: Xanthosine monophosphate
GMP: Guanosine monophosphate
HPRT: Hypoxanthine–guanine phosphoribosyltransferase
APRT: Adenine phosphoribosyltransferase
PRPP: Phosphoribosyl pyrophosphate
PRA: 5-Phosphoribosylamine
cAMP: Cyclic adenosine monophosphate
ADO: Adenosine
P2RX7: P2X7 receptor
ENTPDase: Ectonucleoside triphosphate diphosphate hydrolase
VAM: Vancomycin/ampicillin/metronidazole
PPs: Peyer’s patches
Tfh: T follicle-helper
Tfr: T follicle-regulated
SFB: Segmented filamentous bacteria
Foxp3: Forkhead box protein 3
IPEX: Immune dysregulation, polyendocrinopathy, and enteropathy, with X-linked inheritance
ENT1: Equilibrative nucleoside transporter 1
CNT2: Concentrative nucleoside transporter 2
ICB: Immune checkpoint blockade
